# Supplementary material for: Rab29 activation of the Parkinson's disease‐associated LRRK2 kinase
Source: EMBO J. 2017 Dec 6;37(1):1–18. doi: 10.15252/embj.201798099 (PMC5753036; doi:10.15252/embj.201798099)
Supplement: Supplementary file 1 — Expanded View Figures PDF [file EMBJ-37-1-s001.pdf]

# Expanded View Figures

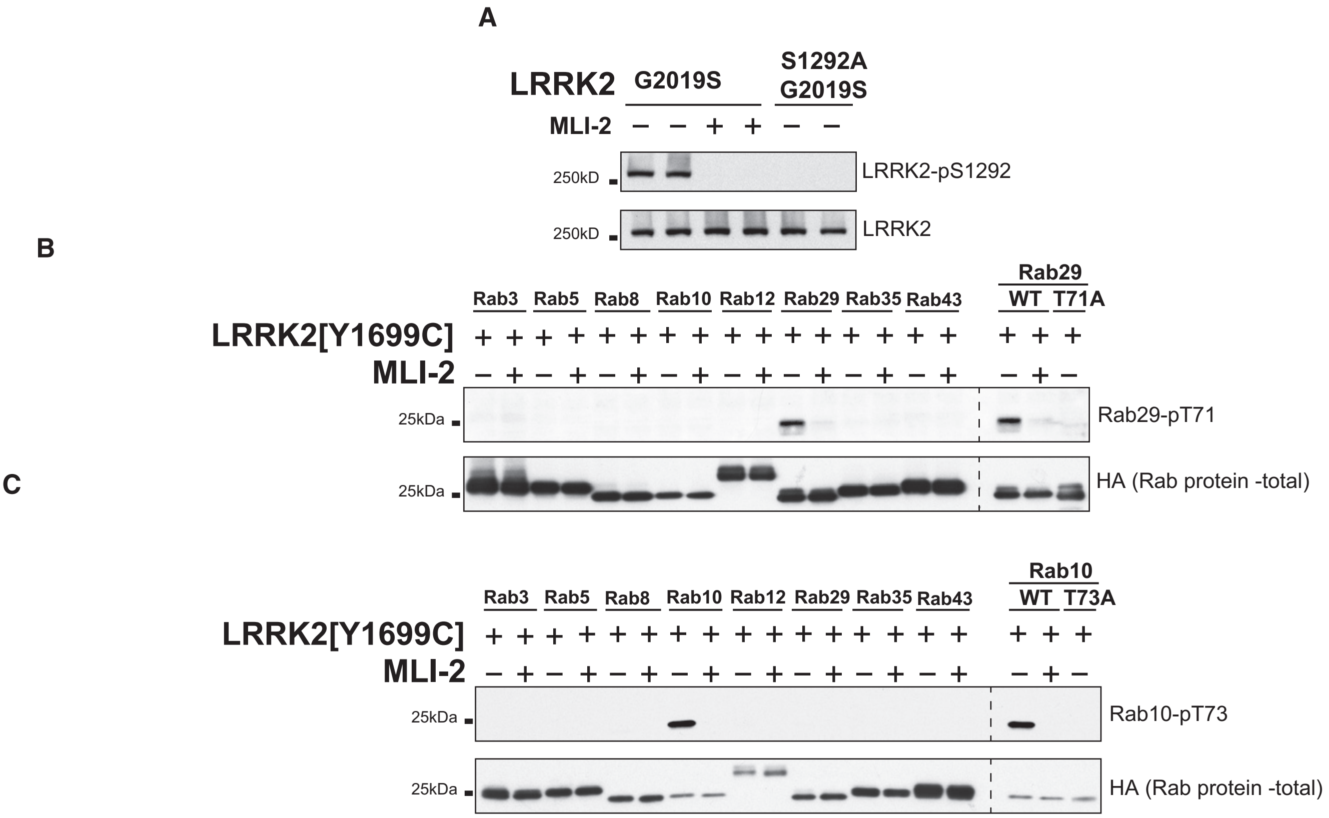


**Figure EV1. Specificity of phospho-antibodies.**

A HEK293 cells were transfected with LRRK2[G2091S] or LRRK2[S1292A, G2019S]. 24 h post-transfection, cells were treated in the presence (+) or absence (−) of the LRRK2 inhibitor MLi-2 (100 nM, 60 min) lysed and analyzed by immunoblotting with the indicated antibodies.

B As in (A) except that the indicated HA-tagged Rab isoforms were co-expressed with LRRK2[Y1699C] in HEK293 cells to maximally phosphorylate these isoforms. Lysates were subjected to immunoblotting with an anti-Rab29 phospho Thr71 antibody.

C Same as (B) except for immunoblotting with an anti-Rab10 phospho Thr73 antibody. No signal is detected upon treatment with MLi-2 or mutation of Rab29 or Rab10 phospho-residue to an alanine. WT, wild type.

© 2017 The Authors

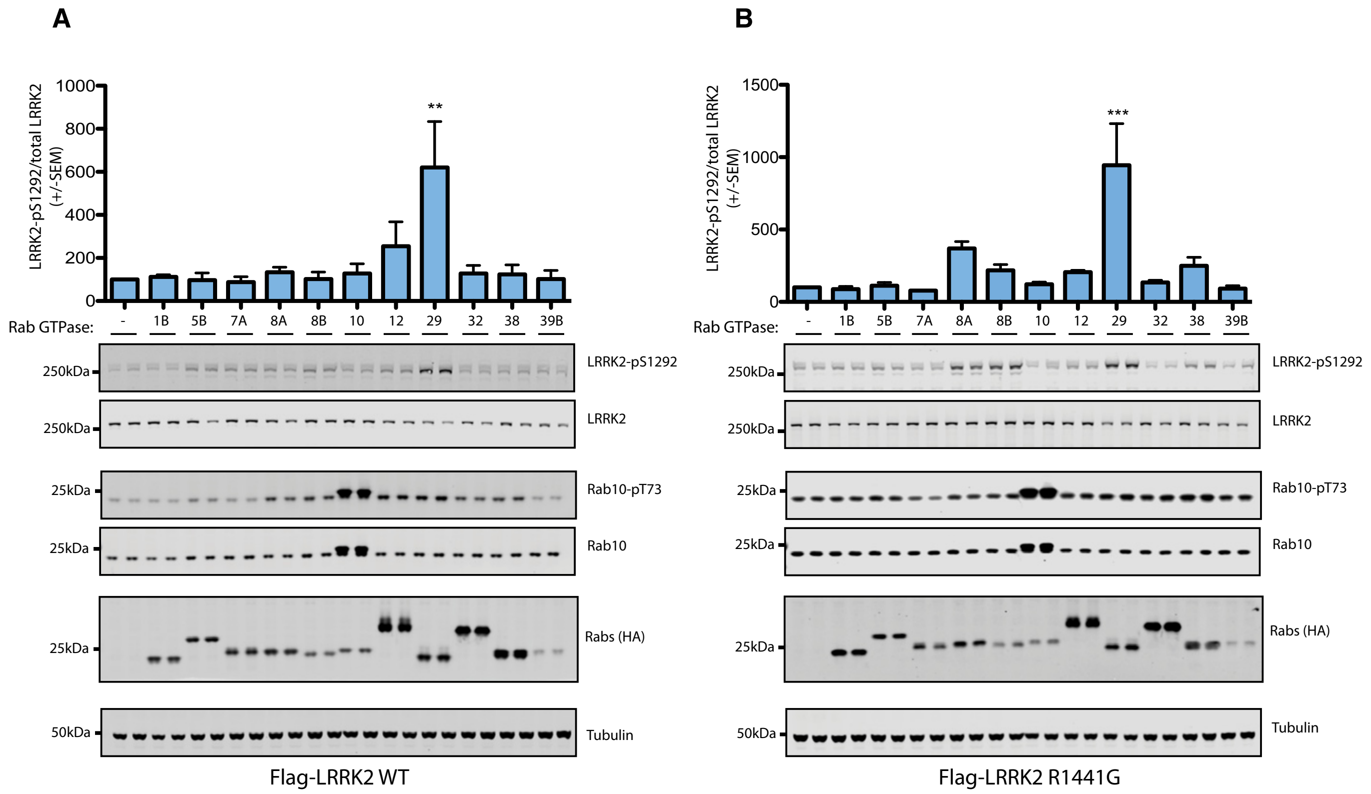


**Figure EV2. Rab29 selectively activates LRRK2.**

A, B HEK293 cells were transfected with the wild-type LRRK2 (A) or LRRK2[R1441G] (B) with either HA-empty vector (–) or the indicated HA-tagged Rab proteins. 24 h post-transfection, cells were lysed and analyzed by immunoblotting with the indicated antibodies. Blots were quantified by LiCor and presented as average ± SEM, and analyzed using one-way ANOVA with Dunnett's multiple comparison test comparing all groups to the HA-empty vector (–). For (A) WT LRRK2, there was a statistically significant difference between groups ($P$ = 0.0135, one-way ANOVA, $F$(11, 12) = 3.911). $^{ns}P > 0.05$, $^{**}P < 0.01$ by one-way ANOVA with Dunnett's multiple comparison test with mean difference 95% confidence intervals of groups compared to HA-empty vector control: Rab1B −356.9 to 332.6; Rab5B −342.0 to 347.6; Rab7A −332.4 to 357.2; Rab8A −378.6 to 311.0; Rab8B −346.7 to 342.9; Rab10 −372.3 to 317.3; Rab12 −499.8 to 189.8; Rab29 −865.3 to −175.7; Rab32 −372.7 to 316.8; Rab38 −367.4 to 322.2; Rab39B −346.5 to 343.1. For (B) LRRK2[R1441G], there was also a statistically significant difference between groups ($P$ = 0.0007, one-way ANOVA, $F$(11, 12) = 7.642). $^{ns}P > 0.05$, $^{***}P < 0.001$ by one-way ANOVA with Dunnett's multiple comparison test with mean difference 95% confidence intervals of groups compared to HA-empty vector control: Rab1B −386.5 to 411.2; Rab5B −410.2 to 387.5; Rab7A −377.0 to 420.7; Rab8A −668.2 to 129.6; Rab8B −517.5 to 280.3; Rab10 −421.1 to 376.7; Rab12 −503.2 to 294.6; Rab29 −1,243 to −445.7; Rab32 −433.5 to 364.3; Rab38 −548.2 to 249.5; Rab39B −390.4 to 407.4. Results were obtained in two separate experiments, each performed in duplicate.

 © 2017 The Authors

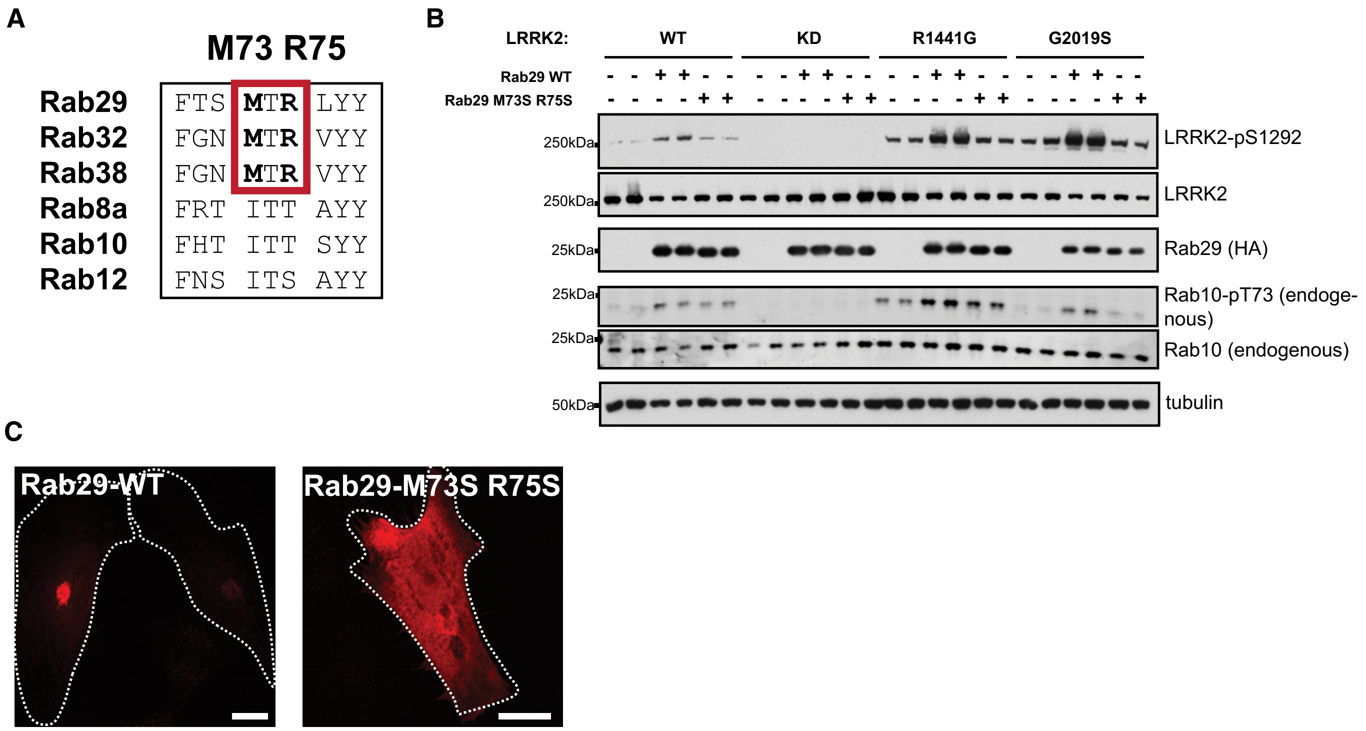


**Figure EV3. Investigation of Rab29 mutations that potentially interrupt Rab29 binding to LRRK2.**

A Sequence alignment of the indicated Rab protein Switch II effector motifs. The MTR sequence that mediates interaction of Rab32 with the ankyrin domain of VARP is highlighted in a red box.

B HEK293 cells were transfected with the indicated wild-type and mutant forms of LRRK2 with either HA-empty vector (–) or HA-tagged wild-type Rab29 (WT) or HA-tagged mutant Rab29[M73S,R75S]. 24 h post-transfection, cells were lysed and analyzed by immunoblotting with the indicated antibodies. WT is wild-type and KD corresponds to the kinase dead [D2017A] LRRK2 mutant. Similar results were obtained in two experiments.

C HeLa cells expressing HA-tagged Rab29 WT or M73S R75S proteins, stained with mouse anti-HA antibody, and analyzed by immunofluorescence microscopy. Scale bars represent 10 μm.

© 2017 The Authors
