# Supplementary figures and images for: Rab29 activation of the Parkinson's disease‐associated LRRK2 kinase
Source: EMBO J. 2017 Dec 6;37(1):1–18. doi: 10.15252/embj.201798099 (PMC5753036; doi:10.15252/embj.201798099)

# Figure 5

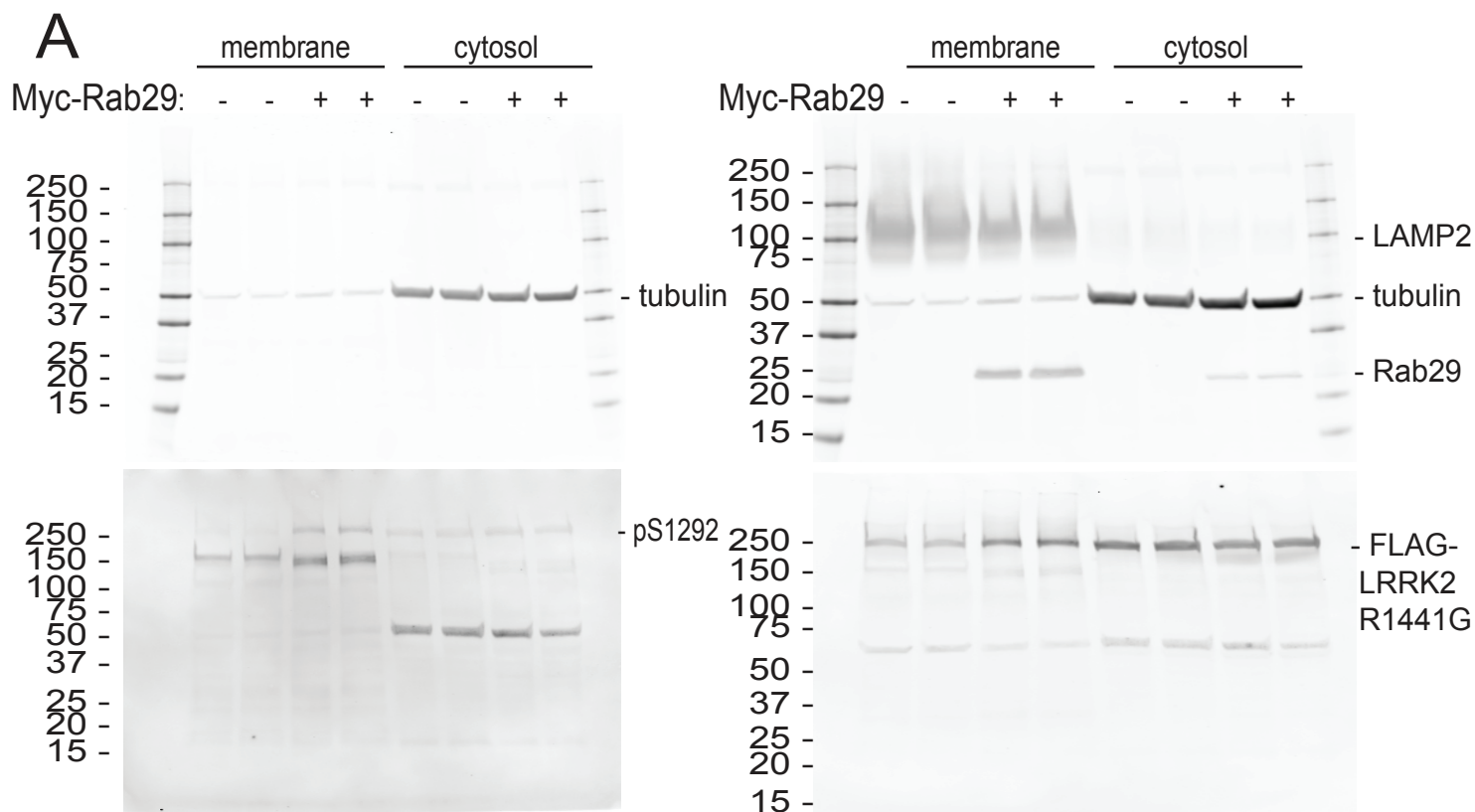

Supplement: Supplementary file 3 — Source Data for Figure 5 [file EMBJ-37-1-s002.pdf]

Source data - Figure 6

Fig6A

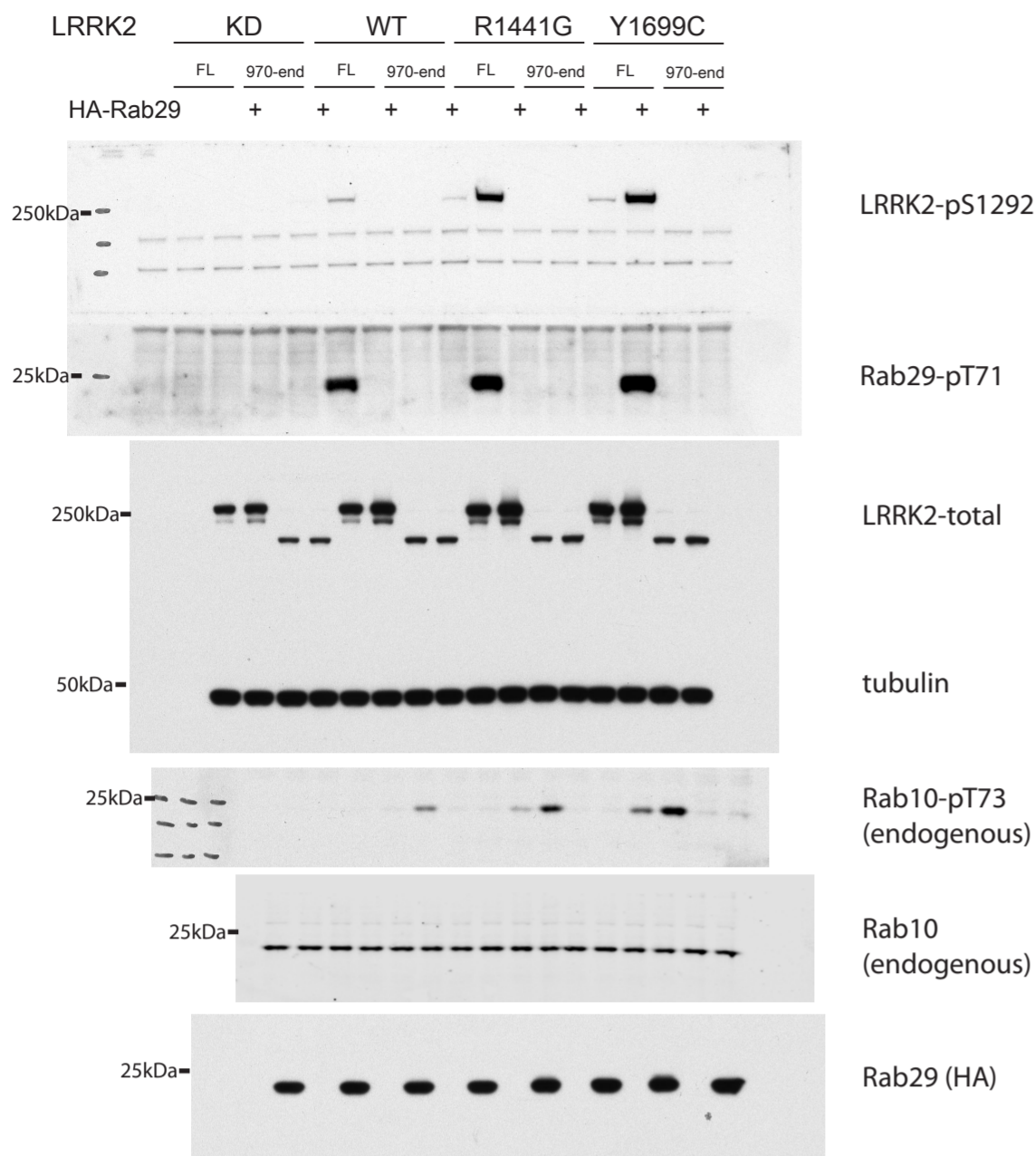

Fig6D

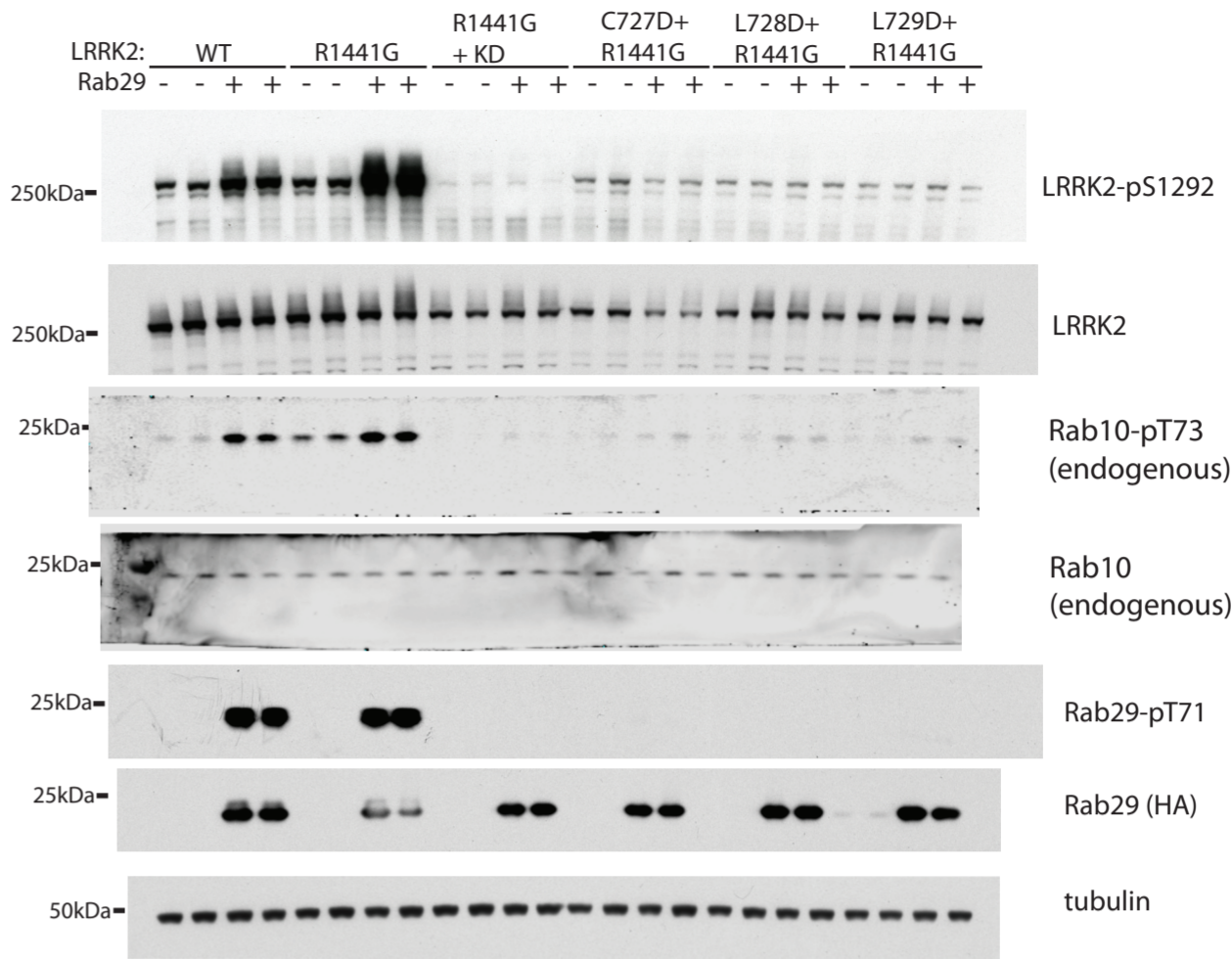

Fig6C

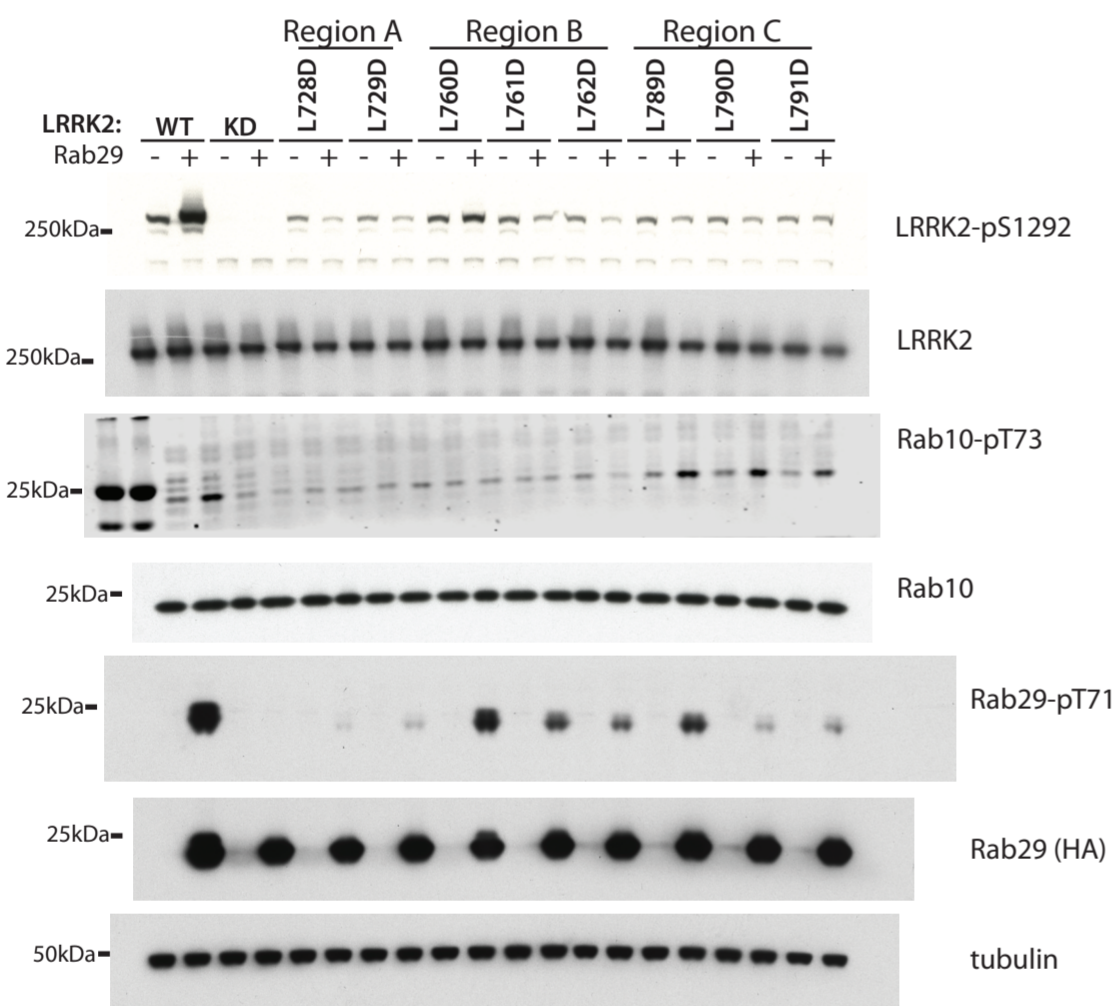

Fig6E

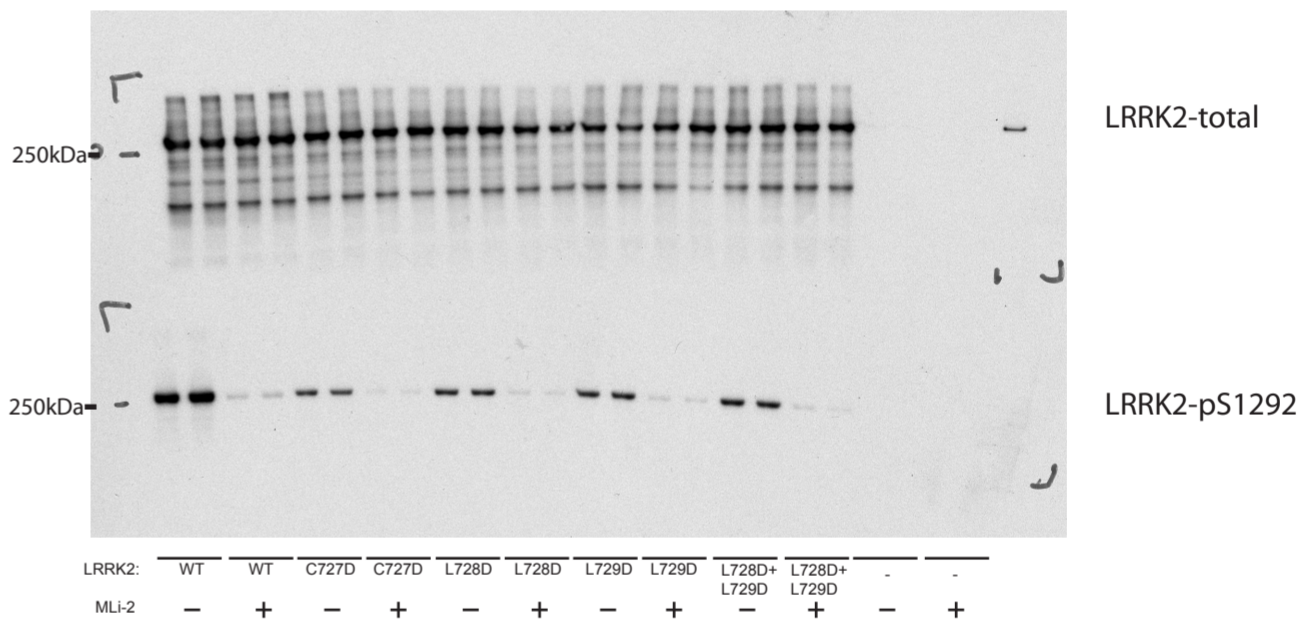

Fig6F

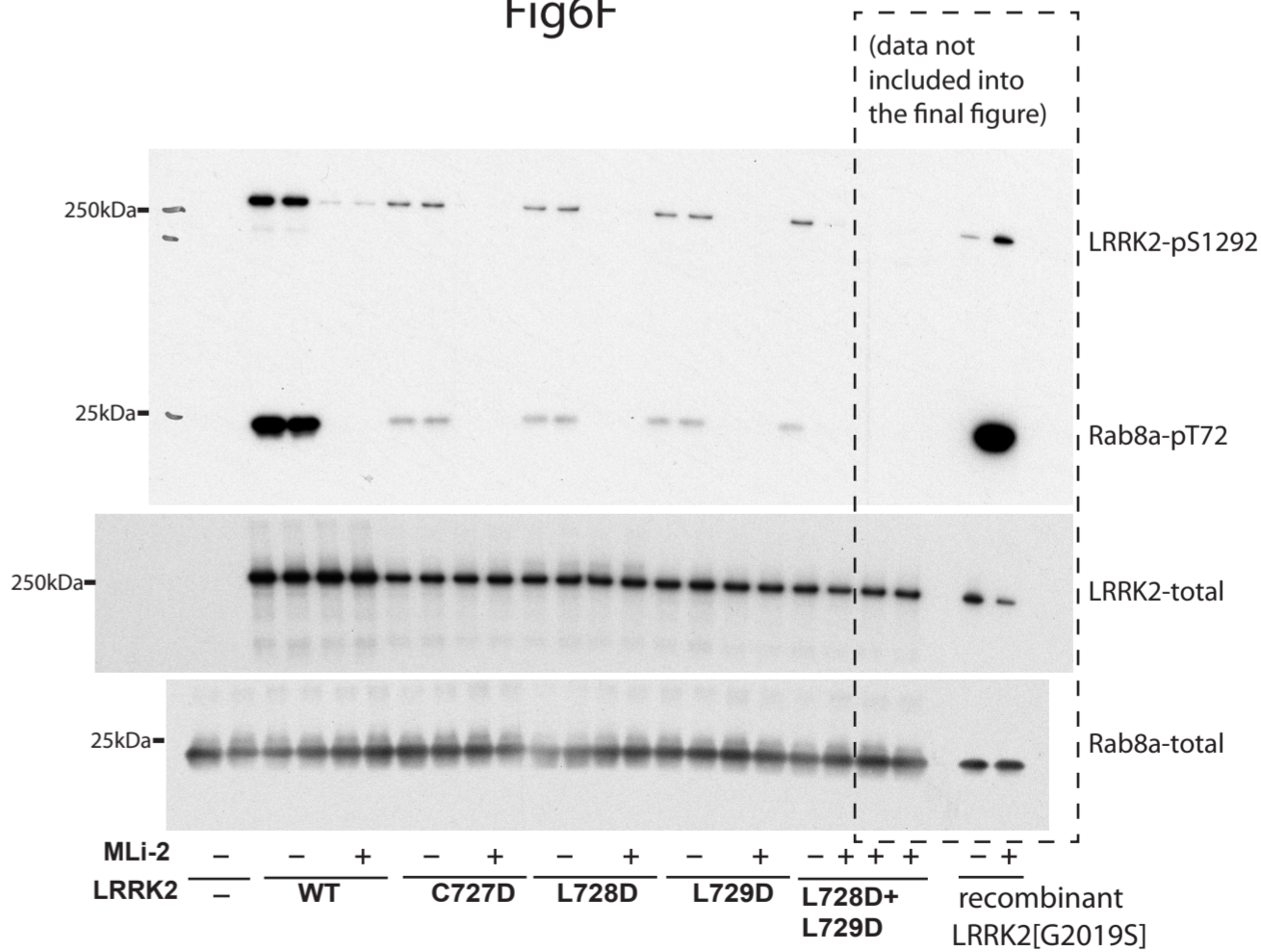

Supplement: Supplementary file 4 — Source Data for Figure 6 [file EMBJ-37-1-s003.pdf]

Figure 7

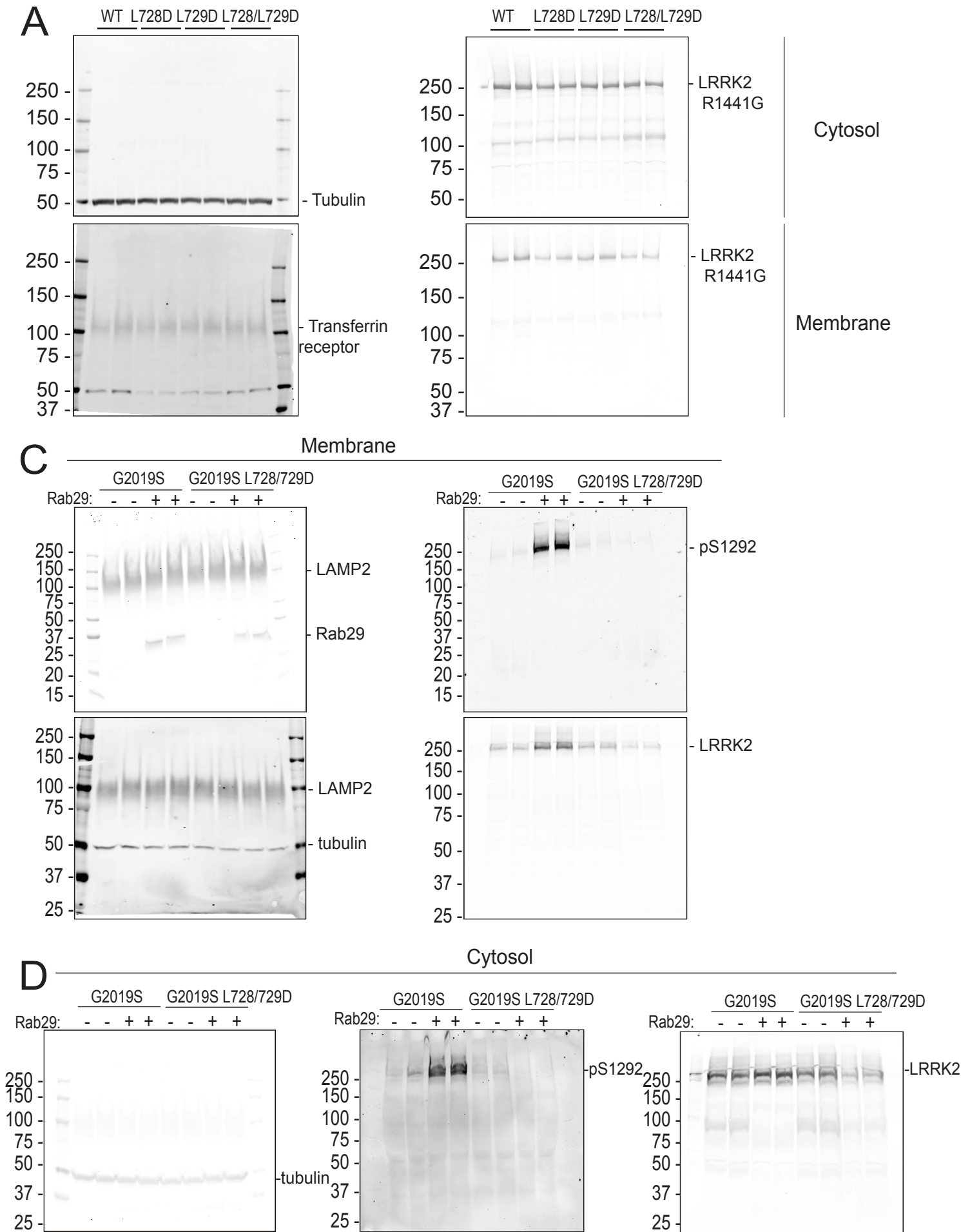

Supplement: Supplementary file 5 — Source Data for Figure 7 [file EMBJ-37-1-s004.pdf]

Source data - Figure 9

Fig9A

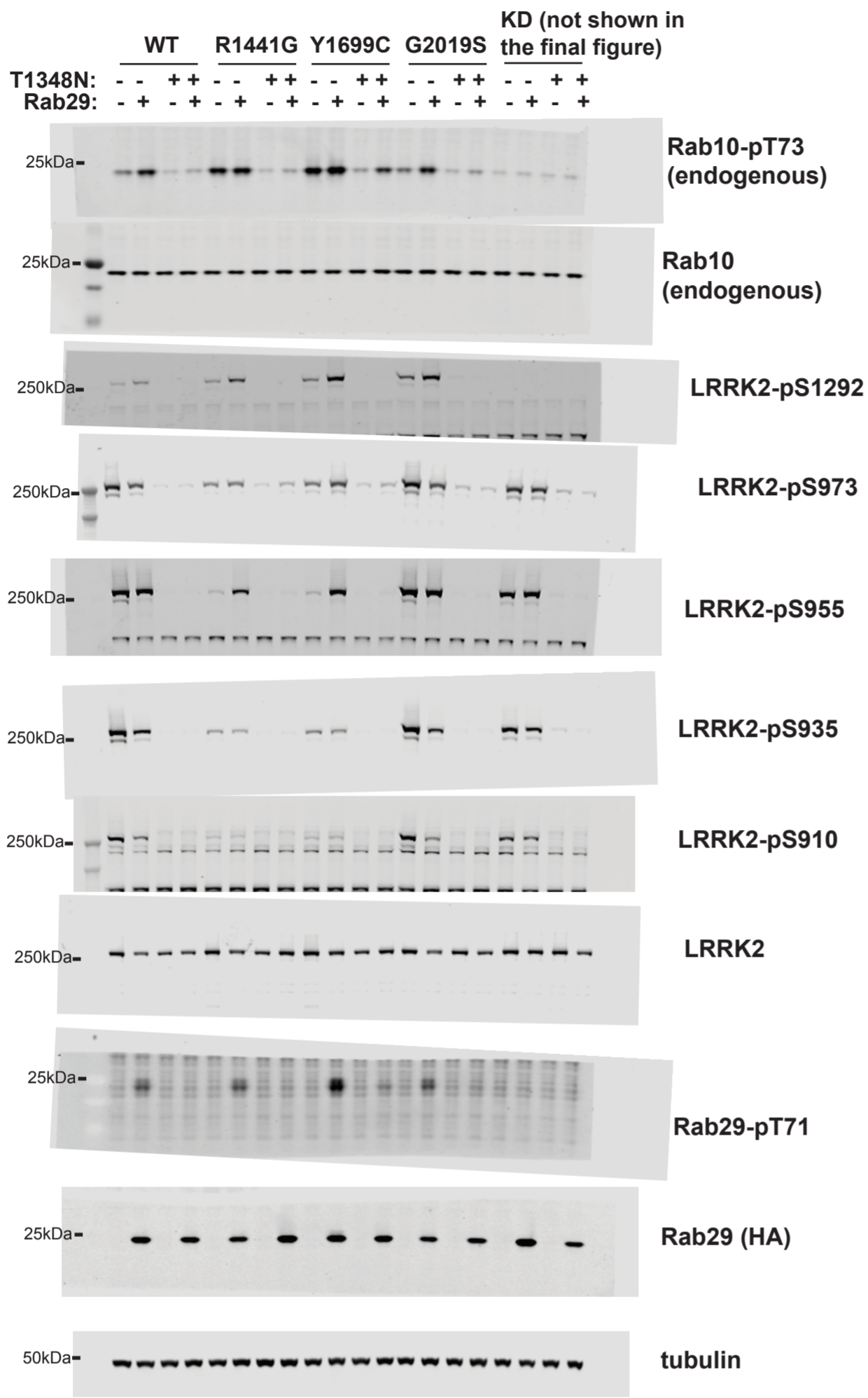

Fig9B

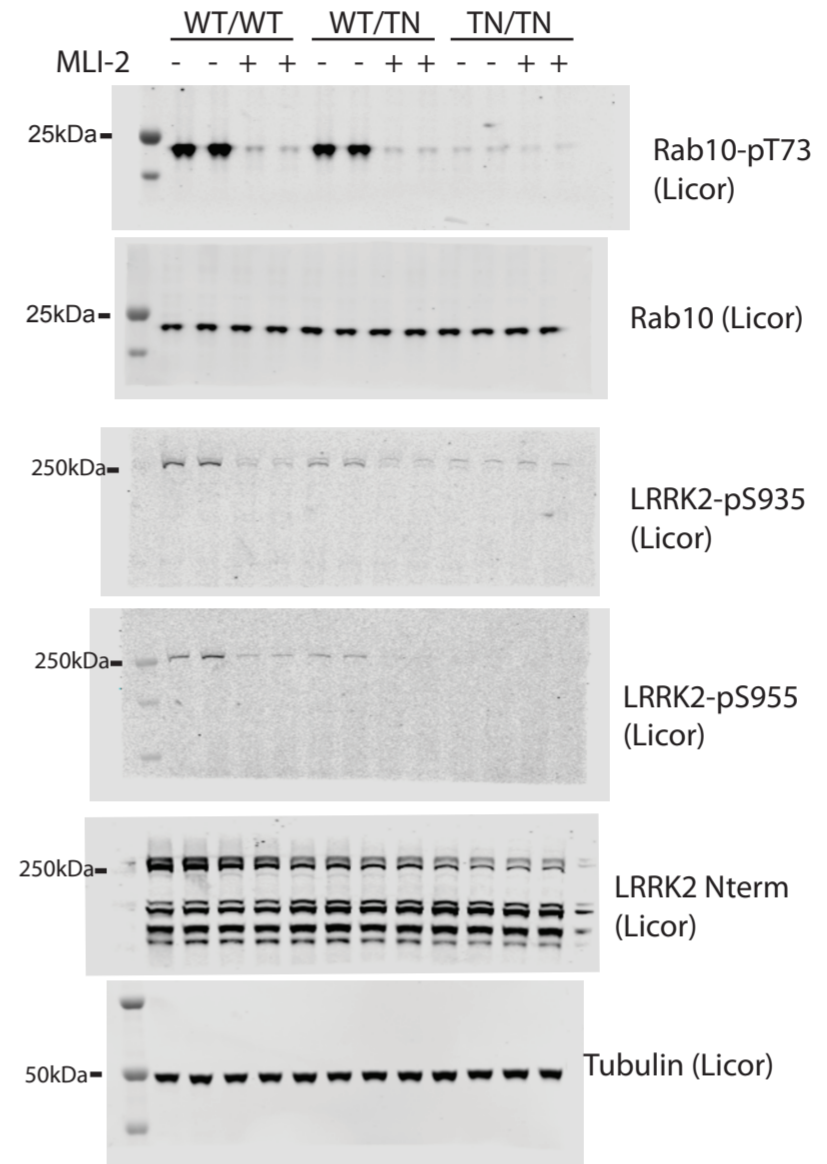

Fig9D

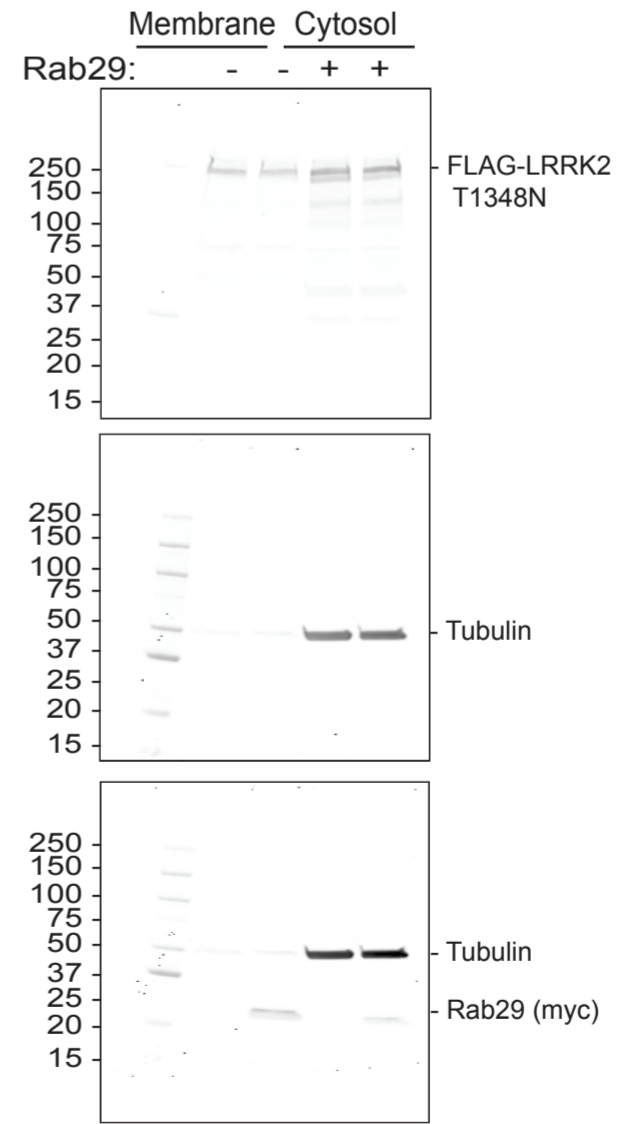

Supplement: Supplementary file 6 — Source Data for Figure 9 [file EMBJ-37-1-s005.pdf]
